# Novel Insights Into the Hyperaccumulation Syndrome in *Pycnandra* (Sapotaceae)

**DOI:** 10.3389/fpls.2020.559059

**Published:** 2020-09-09

**Authors:** Sandrine Isnard, Laurent L’Huillier, Adrian L. D. Paul, Jérôme Munzinger, Bruno Fogliani, Guillaume Echevarria, Peter D. Erskine, Vidiro Gei, Tanguy Jaffré, Antony van der Ent

**Affiliations:** ^1^AMAP, Université Montpellier, IRD, CIRAD CNRS, INRAE, Montpellier, France; ^2^AMAP, IRD, Herbier de Nouvelle-Calédonie, Nouméa, New Caledonia; ^3^Institut Agronomique néo-Calédonien (IAC), Equipe ARBOREAL (AgricultuRe BiOdiveRsité Et vAlorisation), Paita, New Caledonia; ^4^Centre for Mined Land Rehabilitation, Sustainable Minerals Institute, The University of Queensland, St Lucia, QLD, Australia; ^5^Institute of Exact and Applied Sciences (ISEA), Université de la Nouvelle-Calédonie, Nouméa, New Caledonia; ^6^Université de Lorraine – INRAE, Laboratoire Sols et Environnement, Vandoeuvre-lès-Nancy, France

**Keywords:** hydroponic, hyperaccumulation, laticifers, nickel, Pycnandra, X-ray fluorescence spectroscopy

## Abstract

The discovery of nickel hyperaccumulation, in *Pycnandra acuminata*, was the start of a global quest in this fascinating phenomenon. Despite recent advances in the physiology and molecular genetics of hyperaccumulation, the mechanisms and tolerance of Ni accumulation in the most extreme example reported to date, *P. acuminata*, remains enigmatic. We conducted a hydroponic experiment to establish Ni tolerance levels and translocation patterns in roots and shoots of *P. acuminata*, and analyzed elemental partitioning to gain insights into Ni regulation. We combined a phylogeny and foliar Ni concentrations to assess the incidence of hyperaccumulation within the genus *Pycnandra*. Hydroponic dosing experiments revealed that *P. acuminata* can resist extreme Ni concentrations in solution (up to 3,000 µM), and dosing at 100 µM Ni was beneficial to growth. All plant parts were highly enriched in Ni, but the latex had extreme Ni concentrations (124,000 µg g^−1^). Hyperaccumulation evolved independently in only two subgenera and five species of the genus *Pycnandra*. The extremely high level of Ni tolerance is posited to derive from the unique properties of laticifers. The evolutionary and ecological significance of Ni hyperaccumulation in *Pycnandra* is discussed in light of these findings. We suggest that Ni-rich laticifers might be more widespread in the plant kingdom and that more investigation is warranted.

## Introduction

**Figure 1:**
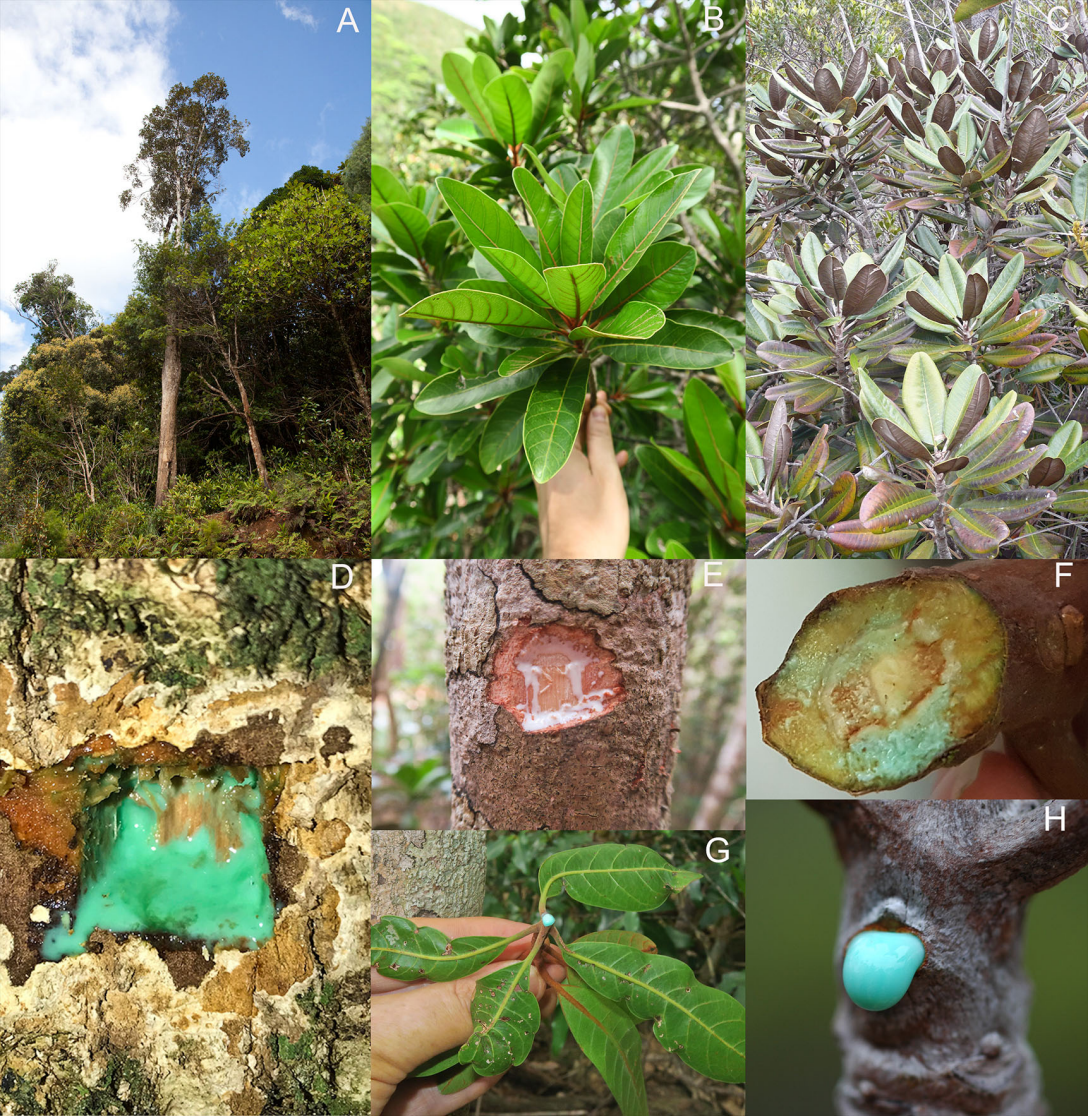
Hyperaccumulator species in the genus *Pycnandra* (Sapotaceae) from New Caledonia. All three species have a blue-green Ni-rich latex. **(A)** Mature specimen of *Pycnandra acuminata* with a tree height of approximately 24 m in the Parc de la Rivière Bleue. **(B)** Individual of *P. caeruleilatex* in Kuebini, Yate. **(C)** Individual of *P. kouakouensis* in Mount Kouakoué. Green-blue Ni-rich latex exuding from the main trunk of *Pycnandra acuminata* **(D)**, white Ni-poor latex exuding from the main trunk of *P. caeruleilatex* **(E)**, green-blue Ni-rich latex exuding a small cut branch of *P. caeruleilatex* **(G)**, green-blue Ni-rich latex exuding a small cut branch and broken petiole of *P. kouakouensis* in **(F–H)**.

The seminal report by Jaffré et al. (1976) on the nickel (Ni)-rich latex of *Pycnandra acuminata* (previously *Sebertia acuminata*; Sapotaceae) introduced the term “hyperaccumulator” and gave rise to a new field of research (Jaffré et al., 1976; Jaffré et al., 2018). Hyperaccumulators are unusual plants that accumulate metals or metalloids (*e.g.* Ni, Co, Mn, Zn) in their living tissues to levels that may be hundreds or thousands of times greater than what is normal for most plants (Reeves, 2003; van der Ent et al., 2013). While most plants only contain ≤10 µg g^−1^ of Ni in their tissues, Ni-hyperaccumulators are capable of accumulating ≥ 1,000 µg g^−1^ of Ni in their tissues (Brooks et al., 1977). The remarkable syndrome of hyperaccumulation is a response to the elevated Ni concentrations typically found in soils derived from ultramafic rocks (*i.e.* Mg- and Fe-rich) (Brooks, 1987). *Pycnandra acuminata*, a large tree endemic to New Caledonia, has attracted the attention of scientists for nearly five decades because of its vivid blue-green latex (**Figures 1A, D**) that contains up to 257,000 µg g^−1^ Ni (Jaffré et al., 1976), the highest Ni concentration ever found in a living organism (Sagner et al., 1998; Rascio and Navari-Izzo, 2011).

Nickel is an essential mineral element for higher plants, even though it is usually required in extremely low concentrations (Brown et al., 1987). One key function of Ni is as an essential component of urease, an enzyme which catalyzes urea hydrolysis for the release of ammonia (Gerendás et al., 1999; Taiz and Zeiger, 2006). This activity contributes to the recycling of endogenous nitrogen for plant growth (Gerendás et al., 1999). In contrast, the exposure of normal plants to elevated Ni concentrations alters the uptake of Fe and Mg provoking chlorosis, and depressing plant growth (Tan et al., 2000; Seregin and Kozhevnikova, 2006; Nishida et al., 2011). The critical toxicity level for Ni in normal plants is about 10 to 50 µg g^−1^ (Krämer, 2010). Nickel hyperaccumulator plant species have extraordinarily high levels of resistance and can tolerate high concentrations of Ni in soil and in solution in cultivation. In hydroponics experiments, the biomass production of hyperaccumulators (*e.g.* *Alyssum bertolonii*, *Noccaea goesingense*, *Berkheya coddii*) remains unaffected by Ni concentrations of up to several hundred µM in the hydroponic solution (Gabbrielli et al., 1991; Krämer et al., 1997; Robinson et al., 2003).

Several hypotheses have been put forth to explain the selective advantage of the hyperaccumulation syndrome, ranging from simple sequestration of toxic metals, physiological benefits, drought stress protection, allelopathic effects and “elemental defence”. However there is no consensus, and hyperaccumulation may have evolved for different reasons in different lineages in the plant kingdom (Martens and Boyd, 1994; Boyd and Martens, 1998; Boyd and Jaffré, 2001; Bhatia et al., 2005). To date, most studies regarding the mechanisms of uptake and tolerance of Ni hyperaccumulation have been limited to few model herbaceous species (*Berkheya* (Asteraceae); *Noccaea* and *Alyssum* (Brassicaceae)). Very little is comparatively known about woody hyperaccumulators that represent a large proportion of the diversity of Ni hyperaccumulators in tropical regions of the world (Reeves et al., 2018).

In most hyperaccumulator plants studied to date Ni is preferentially accumulated in foliar epidermal cells (Leitenmaier and Küpper, 2013). In *P. acuminata*, cryo-scanning electron microscopy with energy-dispersive X-ray spectroscopy (SEM-EDS) has shown that (stem) laticifers are highly Ni-enriched, and laticifers were suggested to constitute the main accumulation location (Sagner et al., 1998). Nickel accumulation in laticiferous plants might be particularly high, with the total Ni content of a single mature *P. acuminata* tree estimated at 5.15 kg (van der Ent and Mulligan, 2015). Because *P. acuminata* grows in forested areas over peridotitic alluvia or colluvia relatively rich in Ni (Jaffré et al., 1976; Perrier et al., 2004), but deprived of Ni-ore potential, bio-accumulation of Ni in *P. acuminata* could represent an additional source of highly enriched Ni fluid. More globally, laticifer cells represent natural biofactories for the production of several types of compounds and chemicals offering multiple commercial possibilities (Castelblanque et al., 2017). Nickel-rich laticifers are not only limited to *P. acuminata* and have also been described from several species belonging to the Euphorbiaceae and Phyllanthaceae (Berazaín et al., 2007; Reeves et al., 2007).

Enhanced levels of metal transport in hyperaccumulators has predominantly been shown to involve long-distance transport in the xylem (Lasat et al., 1996; Lasat et al., 1998; Rascio and Navari-Izzo, 2011), and extreme accumulation in the phloem bundles is described as a common feature of most tropical woody hyperaccumulators (van der Ent et al., 2017). The physiology of laticifer cells still remains a *terra incognita* (Pickard, 2008), and laticiferous hyperaccumulators might bring much insight into unexplored long-distance transport of natural products in plants.

New Caledonia is recognized as a global hotspot for metal hyperaccumulator plants, and the territory offers significant opportunities to broaden our understanding of the mechanisms involved in Ni hyperaccumulation (Jaffré et al., 2013; van der Ent et al., 2015; Isnard et al., 2016). Apart from *P. acuminata*, two other recently described *Pycnandra* species from New Caledonia also have a blue-green Ni-rich latex: *P. kouakouensis* and *P. caeruleilatex* (Swenson and Munzinger, 2016) (**Figure 1**), suggesting that hyperaccumulation is not an isolated phenomenon in the genus.

Discoveries of trace element hyperaccumulator plants have historically required time-consuming destructive chemical analysis of fragments from herbarium specimens (for examples in the New Caledonian context, see Brooks et al. (1977); Jaffré et al. (1979); Kersten et al. (1979)), which severely constrained the collection of large datasets. Recent advances in handheld X-ray fluorescence spectroscopy (XRF) systems have enabled non-destructive analysis of herbarium specimens (McCartha et al., 2019; van der Ent et al., 2019b), and this approach has been successfully applied to assess the incidence of hyperaccumulation in the ultramafic flora of New Caledonia (Gei et al., 2020). This work has permitted mass measurements of tens of thousands of samples in herbaria in a relatively short time span at low-cost (van der Ent et al., 2019a). Another advantage of XRF analysis is that the raw data (*e.g.* energy-dispersive X-ray fluorescence spectra) can be reprocessed when more accurate calibration models become available (van der Ent et al., 2019a).

Here, we combine experimental and field approaches to gain insight into the mechanisms of hyperaccumulation in *P. acuminata*. Nickel dosing hydroponic experiments were undertaken to establish Ni tolerance levels and translocation patterns in the roots and shoots of *P. acuminata*. Considering the extremely high accumulation capacity of this species, tolerance was studied using very high Ni concentrations (up to 3,000 µM Ni in solution), thus differing from previous studies that did not exceed several hundred µM in the hydroponic solution (Gabbrielli et al., 1991; Krämer et al., 1997; Robinson et al., 2003). We measured multi-element concentrations in different plant tissues and transport fluids of plants in nature, to gain insight into Ni accumulation partitioning relative to other essential and non-essential elements. Finally, we undertook a systematic XRF re-assessment of the full *Pycnandra* genus, in combination with a resolved phylogeny, to investigate the phylogenetic incidence of hyperaccumulation in this genus.

## Materials and Methods

### Nickel Dosing Experiment With *Pycnandra acuminata* Seedlings

*Pycnandra acuminata* seeds were germinated at 27°C in vermiculite moistened with demineralized water. After a period of 4 weeks, seedlings with their cotyledons and 2 young leaves were transferred to 5-L containers (4 seedlings per container) containing one-tenth strength modified Hoagland’s solution at pH 5.3 (Ca, 0.25 mM; Mg, 0.06 mM; K, 0.37 mM; NH_4_, 0.12 mM; NO_3_, 0.87 mM; PO_4_, 0.12 mM; SO_4_, 0.06 mM; Cl, 3.1 μM; B, 1.5 μM; Mn, 0.1 μM; Zn, 0.1 μM; Cu, 0.03 μM; Mo, 0.03 μM; Fe-EDTA, 1.2 μM), in order to be close to the natural soil solution (Becquer et al., 2010). The nutrient solutions were supplemented with 0, 100, 300, 1,000, or 3,000 μM Ni as NiSO_4_.6H_2_0. They were continuously aerated and renewed every 7 days. Growth conditions were 25:21 ± 1°C (light (L): dark (D)), 70:80 ± 5% (L:D) relative humidity, 14 h L: 10 h D daily photoperiod, with 200 ± 10 μmol m^−2^ s^−1^ photon flux density at leaf level. A randomized block factorial design with five Ni concentrations, and five replicates was used.

After 180 days of growth under these conditions, the plants were harvested and separated into roots and shoots, rinsed twice in distilled water, dried at 105°C, and finally weighed. *Pycnandra acuminata* fruits are known to contain 3,000 to 5,000 µg g^−1^ Ni (Jaffré et al., 1976; Sagner et al., 1998), with 5,000 µg g^−1^ in the cotyledons (Sagner et al., 1998). Seedlings can translocate Ni from the cotyledons into the shoots during development (Chaney et al., 2009), therefore, shoots were separated into two compartments to distinguish between the portion of stem that grew in the presence of cotyledons (Pre-Cotyledons-Shoots, PrCS) and the younger portion of stems that grew after the fall of the cotyledons (Post-Cotyledons Shoots; PstCS). For this purpose, the position of the last leaves was marked, once cotyledon fell. This sampling procedure was used to assess Ni translocation within the shoot. Shoot length was measured from cotyledon marks to the apical meristem. Plant samples (PrCS, PstCS, roots) were then ground, dry-ashed at 485°C, and further oxidized and re-dissolved in HNO_3_. Elemental concentrations of Ca, K, Mg, P, and Ni were determined by Inductively coupled plasma atomic emission spectroscopy (ICP-AES).

### Field Collection and Chemical Analysis of Plant Tissue Samples

*Pycnandra acuminata* (Baill.) Swenson & Munzinger (formerly *Sebertia acuminata* and known locally as “Sève bleue” tree) is rare and restricted to lowland humid forest on ultramafic soils, mainly in the southern ultramafic massif but also on the east side and in the northwest of the main island (Swenson and Munzinger, 2010).

Plant tissue samples (leaves, twigs, wood, bark, phloem, latex, xylem sap) for bulk chemical analysis were collected in the field, from a population located at the Plaine des Lacs (22°16′27.94”S, 166°54′12.44”E) in the southern massif. Bark and wood samples were taken from small diameter branches (2–3 cm) by stripping the bark with a sharp stainless-steel knife, whereas phloem samples were collected by stripping sections from beneath the bark using a razor blade. Latex samples were obtained by slicing a groove into the bark of the tree (**Figure 1D**). Xylem sap was collected with a handheld vacuum pump from excised branches, after bark removal to prevent contamination by phloem sap.

These samples were dried at 70°C for five days in a drying oven, gamma irradiated and subsequently ground and digested using 4 ml HNO_3_ (70%) in a microwave oven (Milestone Start D) for a 45-minute program. After dilution to 30 ml with ultrapure water (Millipore 18.2 MΩ·cm at 25°C) they were analyzed with ICP-AES with a Thermo Scientific iCAP 7400 instrument for macro-elements (Mg, P, K, Ca) and trace-elements (Mn, Fe, Co, Ni, Zn) in radial and axial modes depending on the element and expected analyte concentration. All elements were calibrated with a 4-point curve covering analyte ranges in the samples. In-line internal addition standardization using yttrium was used to compensate for matrix-based effects.

Additional material from the two other recently described *Pycnandra* species with a blue-green Ni-rich latex: *P. kouakouensis* and *P. caeruleilatex* (Swenson and Munzinger, 2016) (**Figure 1**), were analyzed and presented in **Supplementary Table 1**.

### Herbarium X-Ray Fluorescence Spectroscopy

The foliar elemental concentrations of the herbarium specimens were measured using a Thermo Fisher Scientific Niton XL3t 950 GOLDD+. After the initial large-scale study reported in Gei et al. (2020) was completed, efforts were made to further improve the calibration model for XRF measurements of New Caledonian plants, many of which have atypical morphological characteristics (such as thick coriaceous leaves), which can affect the accuracy of XRF measurements which is “matrix sensitive.” A substantially improved newer calibration was used in this study to re-evaluate the distribution of Ni hyperaccumulation within the Sapotaceae and to finely assess the phylogenetic distribution of Ni hyperaccumulation in the genus *Pycnandra*. This new calibration was obtained from 221 specimens from the Herbarium of New Caledonia (NOU) that were intentionally chosen to cover a very wide concentration range (low range to hyperaccumulation range for Mn, Co, Ni, Zn) on the basis of the earlier study (**Supplementary material**). From each specimen a 1-cm^2^ area was destructively excised from each specimen, analyzed by XRF and after digestion by ICP-AES. The resulting regression equation used for “calibration” were substantially improved (for instance R^2^ improved from 0.87 to 0.98), and the newer calibration was used in this study (**Supplementary Figure 1**).

In total, 2,148 specimens of the Sapotaceae, including 847 specimens of *Pycnandra*, were analyzed. The XRF analysis was undertaken on a sheet of “herbarium cardboard” on a pure titanium plate (~99.995%, 2 mm thick ×10 × 10 cm) to provide a uniform background and block transmitted X-rays. The XRF analysis used the “Soils Mode” in the “Main filter” configuration for 30 s duration (Gei et al., 2020). For each herbarium specimen, one XRF measurement was taken from a mature leaf (**Supplementary Material 1**).

The molecular phylogenetic trees of *Pycnandra* was simplified and adapted from Swenson et al. (2007, 2015), including 57 of the 59 described species, plus 4 undescribed species (*Pycnandra fastuosa* W (=open-veined), P. Munzinger 3385, P. Butaud 3343, P. Munzinger 5673, see Swenson and Munzinger, 2016 for details). *Niemeyera whitei* was used as outgroup (Swenson et al., 2007).

### Data Processing

The matching XRF and ICP-AES data was used to obtain calibration curves. The apparent limits of detection (LOD) were estimated by visual inspection of the log-transformed regression models of the XRF data against corresponding ICP-AES measurements and set at XRF values: 107 μg g^−1^ for Ni (range, 107–113,987, n = 149), 426 μg g^−1^ for Co (372–9,532, n = 50), 455 μg g^−1^ for Mn (455–176,396, n = 159), and 27 μg g^−1^ for Zn (range, 27–1,238, n = 117). The residuals *vs.* fitted values were inspected for each linear regression analysis, and outliers (± 3 SD of the residual) were identified and removed. Secondary linear regression models were then derived after the samples with XRF values below the limit of detection were removed. The regression models (y = calculated ICP-AES; x = measured XRF) are: Ni: y = 0.2351x^1.0969^ (R^2^ 0.98), Mn: y = 0.7869x^0.9165^ (R^2^ 0.98), Co: y = 0.429x^0.9809^ (R^2^ 0.92), and Zn: y = 0.3766x^1.1259^ (R^2^ 0.88) (**Supplementary Figure 1**). The data from the Ni dosing experiment were analyzed by ANOVA after being checked for homogeneity of variance. Significance of differences between means was performed using *t*-test at the 95% confidence limit. The Waller-Duncan k-ratio t-test (α = 0.05) was used to determine the effect of treatments for all the measured parameters in the hydroponics experiment.

## Results

### Nickel Dosing Experiment on *Pycnandra acuminata* Seedlings

**Figure 2:**
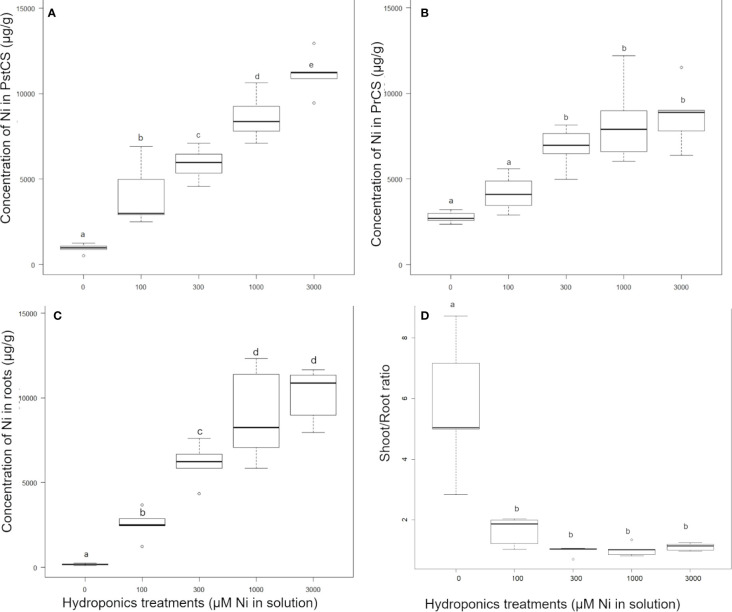
Effect of nickel concentrations in the nutrient solution on the nickel concentration in *Pycnandra acuminata*, after 180 days of growth in hydroponics (n=5). **(A)** PrCS, **(B)** PstCS, **(C)** root and **(D)** shoot to root ratio. Letters indicate significant difference at 5% level (Waller-Duncan k-ratio t-test).

*Pycnandra acuminata* plants were cultivated in hydroponic solutions (spiked with 0, 100, 300, 1,000, or 3,000 µM Ni) for 6 months and growth, biomass production, and elemental concentrations in roots and shoots were analyzed. The total mean Ni concentrations in shoots ranged from ~500 to 12,900 µg g^−1^ across treatments and shoot compartments (**Table 1**). In the control treatment, the plants had translocated Ni stored in the original seed to their shoots, and to a lesser extent in their roots, Ni concentration was consequently strongly compartmentalized as indicated by significantly higher values in the basal part of the shoots (PreCS) followed by PstC and roots (**Table 1**). No significant difference in Ni concentration between the shoot compartments were found in the other treatments, except in the 3,000 µM Ni treatment, where Ni accumulates at significantly higher concentrations in the apical shoot (11,200 µg g^−1^ *vs.* 8,700 µg g^−1^) (**Table 1**). The apical shoot/root concentration ratio (TF) was significantly higher in the control treatment (**Figure 2D**) but did not vary with increasing Ni treatment (from 100 µM to 3,000 µM). TF mean values were around 1 (0.97<TF<1.62) across Ni treatments, meaning that the average Ni concentration in the shoots is (almost) always greater than the corresponding root Ni concentration.

**Table 1:** Nickel concentrations in the different plant compartment (PreC-Shoots, PstC-Shoots and roots) of *Pycnandra acuminata* after 180 days of growth in hydroponics for different Ni concentration in the nutrient solution.

Treatment (µmol L^−1^ Ni)	n	Tissue nickel concentrations		
		PreC-Shoots	PstC-Shoots	Roots
0	5	2,360–3,230 [2,770]a	508–1,260 [948]b	102–250 [176]c
100	5	2,900–5,600 [4,190]a	2,500–6,910 [4,070]a	1,230–3,690 [2,550]a
300	5	4,990–8,160 [6,850]a	4,580–7,100 [5,900]a	4,340–7,620 [6,150]a
1000	5	6,050–12,200 [8,350]a	7,110–10,600 [8,640]a	5,835–12,300 [8,980]a
3000	5	6,390–11,500 [8,730]a	9,460–12,900 [11,200]b	7,970–11,700 [10,200]ab

Letters indicate significant difference between tissues for each treatment, at 5% level (Waller-Duncan k-ratio t-test). All values in in µg g^−1^ dry weight.

Nickel concentrations increased significantly across all treatments in the apical compartment (PstCS) while in the older compartment (PreCS) Ni concentrations were not affected by treatments above 300 µM, and a significant increase was found only between the 100 and 300 µM treatments (**Figures 2A, B**). In the roots, Ni concentrations increased in the 100, 300, and 1,000 µM Ni treatments, but was not affected by treatments above 1,000 µM (**Figure 2C**).

**Figure 3:**
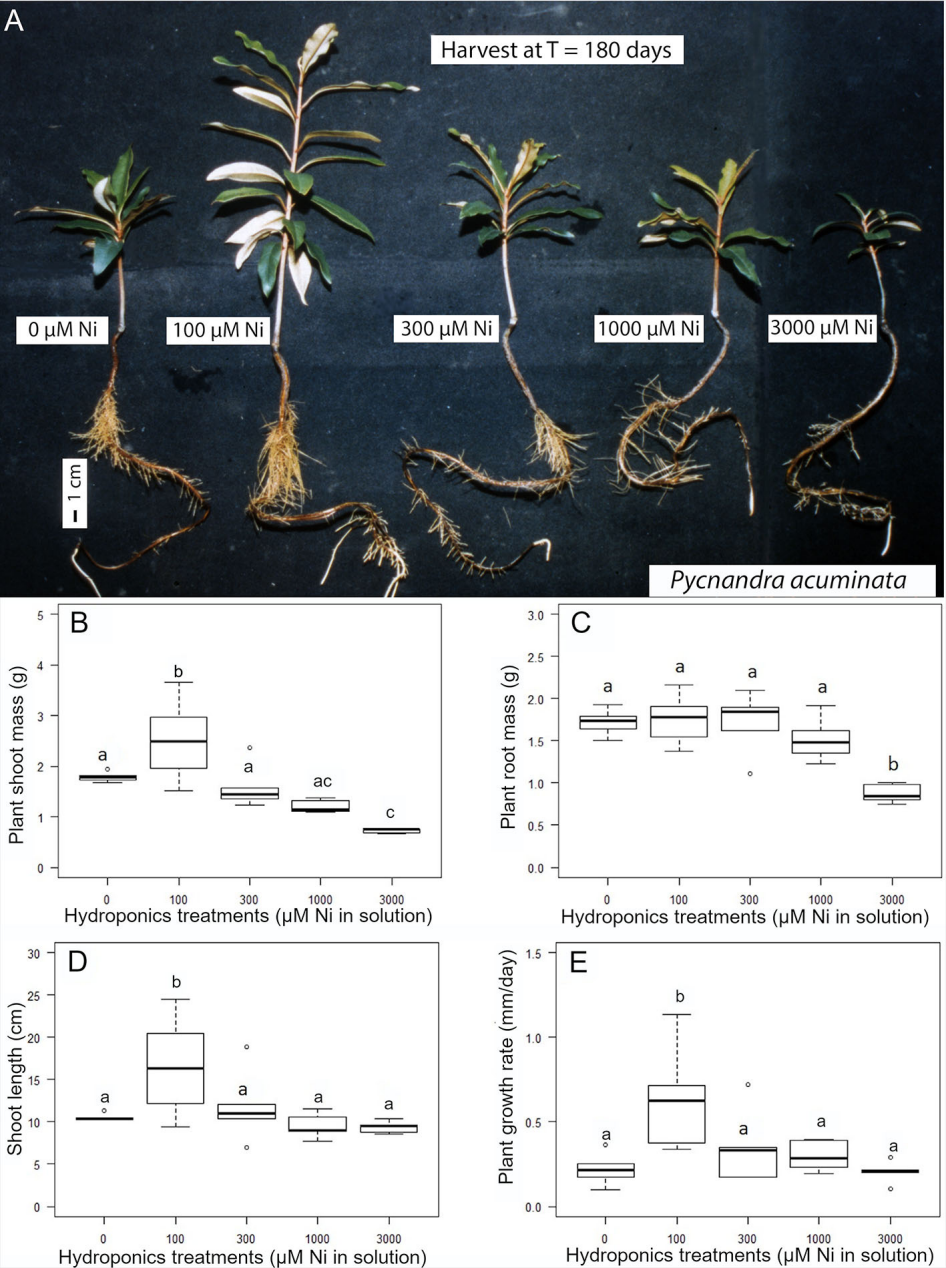
Nickel tolerance of *Pycnandra acuminata* in hydroponic culture. **(A)** Plants exposed to the indicated concentration of Ni for 180 days. **(B–E)** Effect of nickel concentration in the nutrient solution on growth (n = 5). **(B)** Plant shoot biomass, **(C)** plant root mass, **(D)** plant shoot height and **(E)** plant mean relative growth rate. Letters indicate significant difference at 5% level (Waller-Duncan k-ratio t-test).

The exposure of *P. acuminata* to increasing Ni concentrations induced different growth reduction in the shoots and roots (**Figure 3**). Shoots have a higher sensitivity to concentration in the solution above 100 µM Ni, while roots exhibited greater ability to tolerate Ni up to 1,000 µM Ni in the nutrient solution. Only the extreme 3,000 µM Ni treatment significantly impacted the root biomass (**Figure 3C**). Shoot growth was significantly stimulated in the 100 µM Ni treatment (+40% shoot mass), with more individual variation, and similar to the control in the 300 and 1,000 µM Ni treatment (**Figure 5B**). Plant shoot biomass was significantly impacted by Ni in the 3,000 µM Ni treatment (**Figure 3B**), but no symptoms of toxicity, such as chlorosis, were observed across treatments (**Figure 3A**). Similar results were obtained for the shoot length and the daily growth rate although there was no significant difference between the 300, 1,000, and 3,000 µM treatments (**Figures 3D, E**). Macronutrient concentrations in plant parts were not affected by the Ni treatment, although there was a decrease in Ca in the roots and a decrease in Mg concentrations in the different parts of the plants with increasing Ni concentrations in the solution (**Table 2**).

**Table 2:** Bulk elemental concentrations (ICP-AES) of macronutrients in PreC-Shoots, PstC-Shoots, and roots of 180 days *Pycnandra acuminata* plants as a function of the Ni concentration in the nutrient solution.

Treatment (µmol L^−1^ Ni)	Tissue macronutrient concentrations											
	PreC-Shoots				PstC-Shoots				Roots			
	Mg	P	K	Ca	Mg	P	K	Ca	Mg	P	K	Ca
0	1,100	2,400	15,800	5,900	1,700	1,500	25,200	4,000	1,200	4,600	14,100	5,100
100	600	2,500	14,500	6,200	1,000	1,500	22,100	4,500	1,300	4,300	14,500	3,700
300	700	3,000	17,700	5,300	600	1,800	26,200	4,100	800	4,400	13,500	2,300
1000	500	2,700	17,300	3,100	600	1,900	35,600	3,400	600	4,200	12,800	1,200

All values in in µg g^−1^ dry weight.

### Elemental Concentrations in *Pycnandra acuminata*

In *P. acuminata* trees Ni was highly enriched in all parts of the measured, including the leaves, twigs, but exceptionally high Ni was found only in the latex (**Table 3**). The latex had extreme Ni concentrations, reaching 66,000 µg g^−1^ (**Table 3**). The twigs, both bark (including phloem) and wood had high Ni concentrations (up to 11,800 µg g^−1^ Ni in bark). The xylem sap was distinctively Ni-enriched, but the concentration was limited to ~1,430 µg ml^−1^ (**Table 3**). In the leaves, nutrient concentrations (Ca, K, Mg) were relatively high, whereas concentrations of trace-elements were lower (Co, Mn and Zn). All the different plant parts had concentrations below 200 µg g^−1^ and Co was particularly low with no tissue with a mean >15 µg g^−1^. The low Fe concentration indicated that tissues did not have soil particle contamination. Calcium was particularly enriched in the bark (mean 5,690 µg g^−1^) while wood had ~1,500 µg g^−1^ on average. Phosphorus concentrations were low in all plant parts. The latex, besides being strongly enriched in Ni, also had rather high concentrations of Ca, and was Zn enriched compared to other plant tissues. Other elemental concentrations (Mg, P, K, Mn, Fe, Co) were very low in the latex. Compared to Ni, elemental concentration for other elements were also low in the xylem sap.

**Table 3:** Bulk elemental concentrations (ICP-AES) in different plant parts and fluids of *Pycnandra acuminata* (values as means and ranges in µg g^−1^ dry weight).

Plant species	Plant tissue	n	Elemental concentrations								
			Mg	P	K	Ca	Mn	Fe	Co	Ni	Zn
***Pycnandra acuminata***	Old leaves	5	1,560 (1,240–2,440)	276 (244–308)	8,220 (5,800–11,400)	2,660 (1880–3490)	50.8 (30.1–72.8)	137 (34.6–279)	4.77 (0.70–11.0)	6,530 (538–16,900)	43.2 (3.14–109)
	Young leaves	5	2,490 (1,490–3,730)	389 (283–478)	10,700 (8,040–12,400)	3,700 (970–8,400)	72.0 (35.9–145)	80.0 (32.7–139)	10.2 (1.29–37.7)	7,570 (4,260–12,900)	49.8 (24.9–84.4)
	Twigs	5	3,970 (1,060–8,790)	369 (255–489)	9,400 (7,360–13,300)	13,300 (7,550–30,200)	126 (39.8–204)	182 (105–391)	12.6 (1.11–58.1)	10,700 (3,170–14,100)	112 (98.9–139
	Bark (including phloem)	5	666 (441–960)	142 (103–206)	4,670 (3,060–6,890)	5,690 (2,720–12,100)	67.0 (32.7–150)	225 (58.1–349)	2.09 (0.34–4.49)	7,520 (1,280–11,800)	74.4 (9.37–114)
	Wood	5	430 (316–677)	103 (46.2–231)	3,700 (1,780–7,430)	1,610 (548–2,930)	20.1 (11.9–28.0)	24.0 (12.2–36.1)	0.51 (0.14–0.73)	2,930 (1,510–5,180)	26.7 (14.1–49.3)
	Latex	5	177 (121–278)	20.5 (15.9–23.9)	184 (98.2–392)	4,000 (1,310–9,600)	31.1 (13.6–56.6)	23.4 (13.1–31.7)	4.36 (3.78–5.57)	62,800 (57,100–66,300)	367 (330–392)
	Xylem sap (μg ml^−1^)	3	15.4 (9.09–30.7)	0.90 (0.09–1.75)	110 (61.0–160)	124 (71.2–172)	0.85 (0.43–1.74)	0.62 (0.27–0.81)	0.08 (0.03–0.18)	813 (313–1,426)	4.87 (2.00–7.83)

In *P. kouakouensis* another “blue-latex” hyperaccumulator species, the Ni concentrations in leaves were not particularly high (mean value, 3,610 μg g^−1^) but the latex had extreme Ni concentrations (124,000 µg g^−1^) (**Supplementary Table 1**). In *P. caeruleilatex*, Ni concentrations were relatively low with the highest value in the apical tip (2,470 μg g^−1^).

### Incidence of Hyperaccumulation in the New Caledonian Sapotaceae

Herbarium X-ray fluorescence (XRF) scanning was undertaken on all specimens of the Sapotaceae (covering 6 genera) held at the Herbarium of New Caledonia (NOU), totaling 2,148 specimens. The holdings included all genera occurring in New Caledonia, *Manilkara* (1 species), *Mimusops* (1 species), *Pichonia* (7 species), *Planchonella* (36 species), *Pleioluma* (17 species), and *Pycnandra* (62 taxa). Hyperaccumulation of Ni was found with exceedances of the nominal Ni hyperaccumulation threshold (>1,000 µg g^−1^) in the genera *Pichonia, Planchonella* and *Pycnandra* (**Supplementary Figure 2**). In the two former genera, only one to two specimens exceeded this threshold, while in *Pycnandra* many specimens exhibited exceptional [Ni] values. None of the Sapotaceae specimens had a [Mn] above the hyperaccumulation threshold (>10,000 µg g^−1^, (Baker and Brooks, 1989)) (**Supplementary Figure 2**). Values exceeding Zn hyperaccumulation threshold (>3,000 µg g^−1^, (van der Ent et al., 2013)) were recorded in only two specimens of *Pycnandra petiolata* out of 47 specimens (**Supplementary Figure 1**). None of the measured specimens had Co concentration values above the limit of detection.

**Figure 4:**
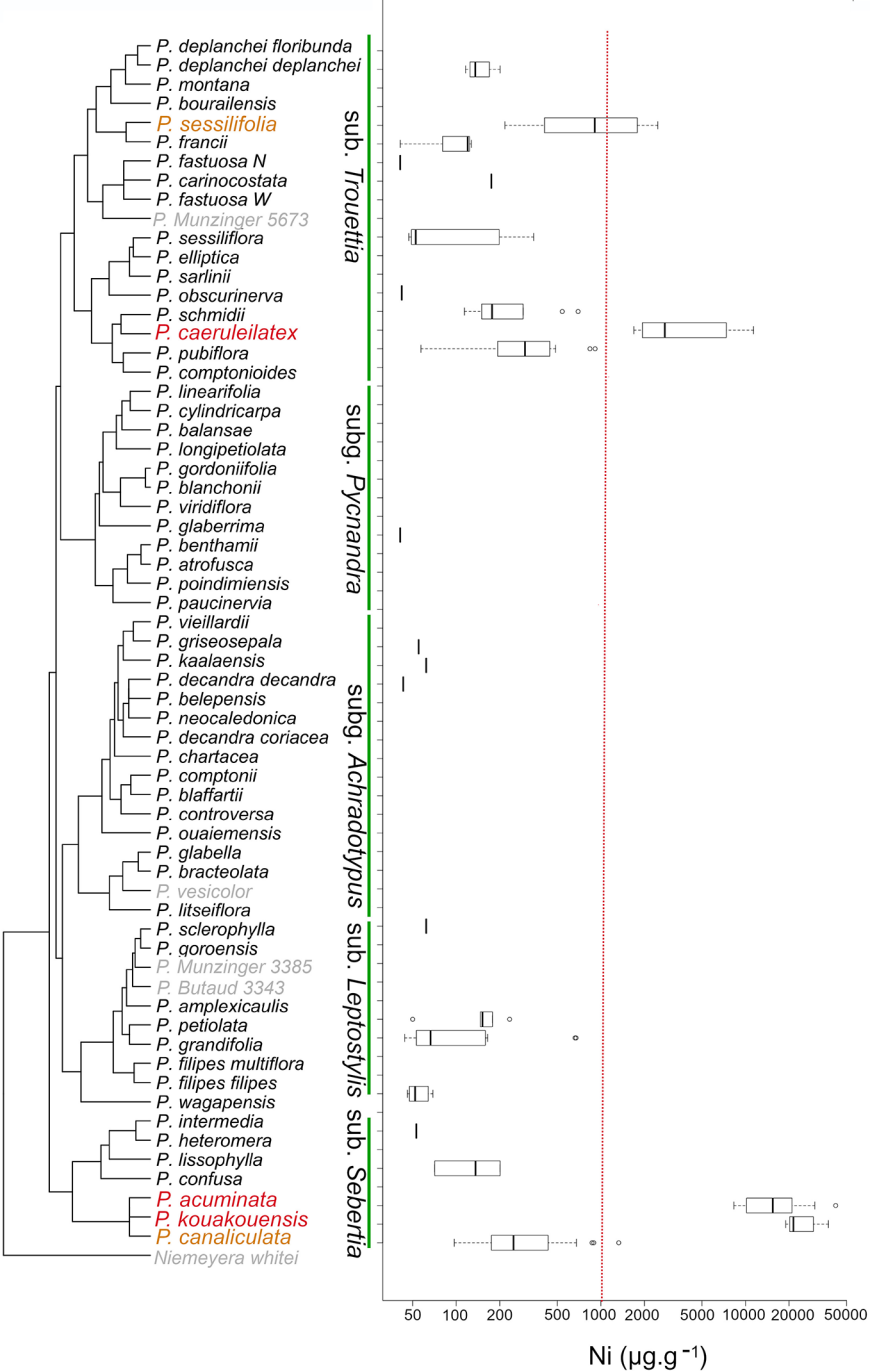
Phylogenetic distribution of hyperaccumulation in the endemic genus *Pycnandra* (Sapotaceae) from New Caledonia. On the left, phylogenetic tree of *Pycnandra*, simplified and adapted from Swenson et al. (Swenson et al., 2007; Swenson et al., 2015), including 57 of the 59 described species, plus 4 undescribed species (*Pycnandra fastuosa* W (=open-veined*)*, P. Munzinger 3385, P. Butaud 3343, P. Munzinger 5673, see Swenson and Munzinger, 2016 for details). Subgenera are indicated vertically, *Niemeyera whitei* is kept as outgroup. On the right, boxplot of [Ni] for each species, based on XRF measurements. Species unavailable for XRF measurements are indicate in grey color. The red vertical dash-line indicates the hyperaccumulation threshold ([Ni] >1,000 µg g^−1^).

Following the XRF assessment, we focused on Ni hyperaccumulation in the genus *Pycnandra*. For most species, concentration values did not exceed the limit of detection, and few species had Ni concentrations around few hundred µg g^−1^ (**Figure 4**). Only five species had incidences of Ni hyperaccumulation: *P. acuminata, P. caeruleilatex, P. canaliculata, P. kouakouensis*, and *P. sessilifolia* (**Figure 4**). For the three blue-green latex species, *P. acuminata, P. caeruleilatex*, and *P. kouakouensis* all specimens exceeded the Ni hyperaccumulation threshold. These species belong to different clades (two subgenus *Trouettia* and *Sebertia*). *Pycnandra acuminata, P. kouakouensis* and *P. canalicula* belong to the same, through unresolved, clade. The highest median foliar Ni concentrations were recorded in *P. acuminata* with 42,000 µg g^−1^ Ni (14 out of 18 specimens had a [Ni] >10,000 µg g^−1^) and *P. kouakouensis* with values between 18,900 and 37,400 µg g^−1^ Ni in the three measured specimens. *Pycnandra caeruleilatex* also reached high [Ni] (up to 11,300 µg g^−1^ Ni) with the four measured specimens exceeding the nominal Ni hyperaccumulation threshold. Almost 50% of the measured specimens of *P. sessiliflora* had foliar concentrations above the hyperaccumulation threshold, but values did not exceed 2,500 µg g^−1^ Ni. The last species, *P. canaliculata*, is a marginal hyperaccumulator, with only one specimen (out of 28) exceeding the hyperaccumulation threshold. While foliar Ni concentrations measured by XRF in herbarium specimens are commonly high for hyperaccumulators species (median, 15,400 µg g^−1^; maximum, 42,000 µg g^−1^ in *P. acuminata*), the bulk foliar Ni concentration did not exceed 16,900 µg g^−1^ in our field collected leaves (**Table 3**) (while up to 25,000 µg g^-1^ has been reported in the literature, Jaffré et al., 2013). This is due to the high sensitivity of XRF measurement and the leaf morphology of *Pycnandra*, as Ni is highly concentrated in the dense laticifer network.

## Discussion

### Nickel Tolerance and Translocation Throughout the Plant

In this study we report extremely high levels of Ni tolerance in cultivated *P. acuminata* plants that did not show any phytotoxicity symptoms when exposed to Ni concentrations as high as 1,000 µM in solution for a long-time (180 days). The concentrations in the growth medium were much more elevated than in experiments on other Ni hyperaccumulator plants (*e.g.* *Noccaea*, *Alyssum*) where Ni concentrations did not exceed several hundred µM in hydroponic solution (Gabbrielli et al., 1991; Krämer et al., 1997; Robinson et al., 2003). The decrease of root biomass suggests the first visible symptom of metal toxicity (Kahle, 1993; Shah et al., 2010), but is significant only in the 3,000 µM treatment. No other obvious toxicity symptoms (*i.e.* no interveinal chlorosis) were observed at concentrations as high as 3,000 µM and Ni continued to accumulate in the apical shoots up to 12,000 µg g^−1^, although the growth of both root and shoots was reduced. Experimental levels of Ni accumulation were similar to concentrations measured in leaves or twigs of trees collected in the field.

**Figure 5:**
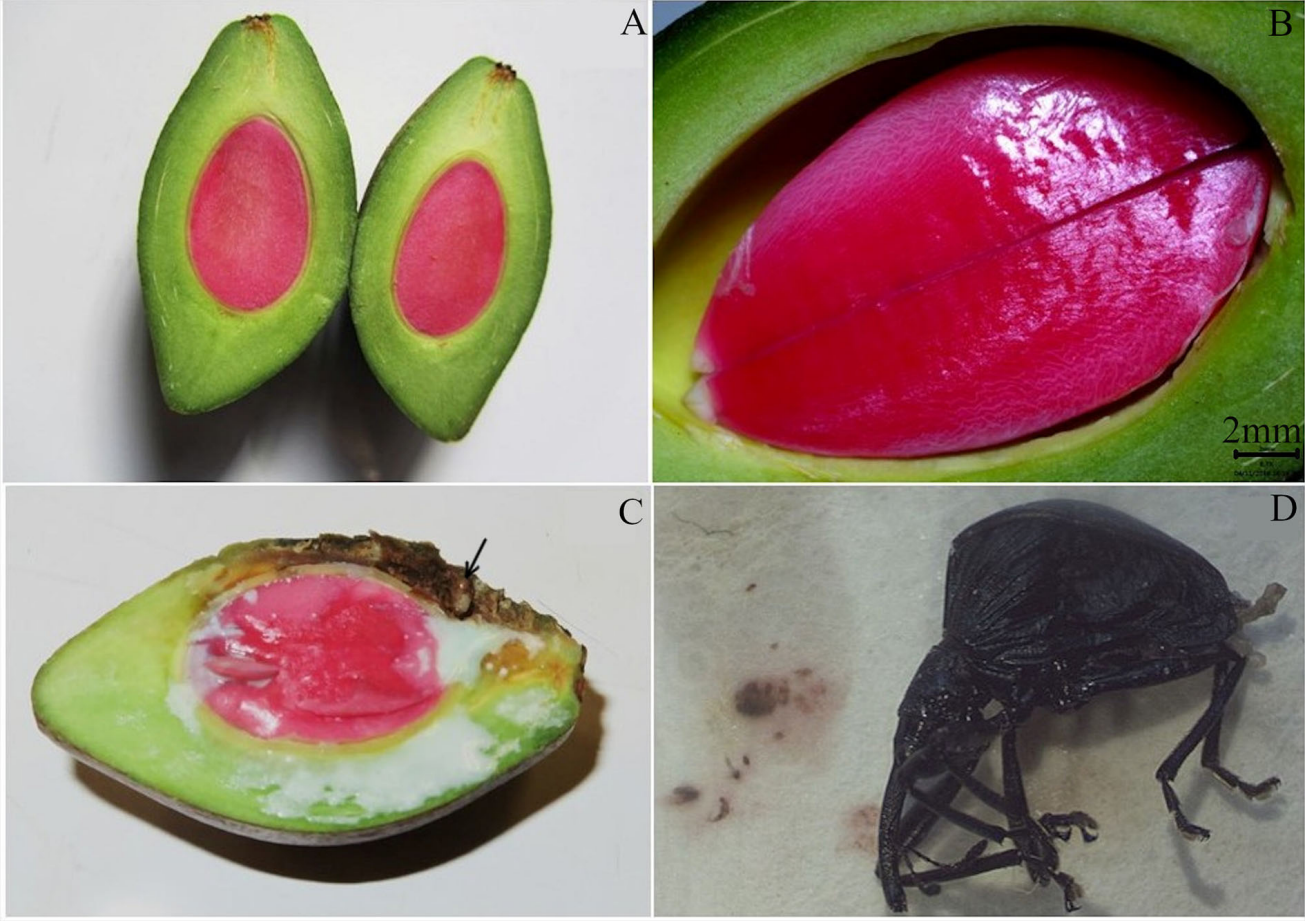
Cut fruit of *Pycnandra acuminata* **(A)**, showing detail of the seed, note laticifer network visible in the cotyledons **(B)**, and **(C)** exuding latex and Apionidae larva (black arrow). **(D)** Adult Apionidae that developed within the fruit. The pressed insect had a positive dimethylglyoxime impregnated-paper staining (red/brown stained), revealing it high Ni content (>1,000 µg g^−1^).

As a consequence of the high endogenous Ni content reported from *P. acuminata* seeds (up to 5,000 µg g^−1^ Ni in the cotyledons, Jaffré et al., 1976; Sagner et al., 1998) (**Figure 5**), foliar Ni concentrations were high in plants of the control treatment (the solution contained at most nanomolar concentrations of Ni). The relatively high Ni concentrations in the shoots and to a lesser extent in the roots of plants, 180 days after germination, is due to translocation of Ni from the seed store. Seed Ni supply has also been reported in another hyperaccumulator species and can provide the young plant with enough Ni to meet the potentially higher Ni requirement of hyperaccumulators (Chaney et al., 2009). The precise location of Ni within the seed tissue of *P. acuminata* is unclear, but bulk analyses of fruits showed that Ni was found in high concentrations in the cotyledons (5,000 µg g^−1^) while concentrations in the embryo were below the detection limit (Sagner et al., 1998).

Laticifers, where most of the Ni is stored in *P. acuminata* (Sagner et al., 1998), are specialized cells known to occur in immature embryos, at the base of emerging cotyledons (Evert, 2006; Castelblanque et al., 2016). It can therefore be reasonably assumed that the laticifers cells in the cotyledons contain a large fraction of the Ni in the seedling (**Figure 5**), and that Ni is transferred from cotyledons to leaves after germination. In our control experiment, Ni in seedlings was preferentially accumulated in old parts of the shoots (PreCS) in comparison to the later developed apical compartment (PstC) (*i.e.* the shoot that developed after cotyledons fall) and root. This Ni partitioning can be explained by the high sequestration function of leaves (laticifers) in *P. acuminata*. Nickel in seedlings is first accumulated in the shoot that develops in the presence of cotyledons, only a small fraction of Ni was transferred to the shoot that developed after cotyledon fall. Increasing Ni in the solution tends to increase Ni concentrations in both shoot compartments and roots, a common physiology of hyperaccumulators plants (Shah et al., 2010). A saturation threshold was reached in the roots and basal part of the shoots (above 1,000 µM Ni treatment), but not in the more recently developed shoots (PstCS), where Ni concentrations continued to increase in the 3,000 µM treatment. This probably reflects a saturation of sequestration capacity of old leaves and roots, and the dynamics of metal uptake and transport that depends on the concentration in the external medium (Gabbrielli et al., 1991; Shah et al., 2010). Note that this also reveals the high sequestration capacity of the roots (up to ~12,000 µg g^−1^), that we infer to be linked to storage in laticifers. Whilst the capacity to translocate and accumulate more metal in the shoots than in the roots is often used as a criteria to identify hyperaccumulator plants (Baker et al., 1994), we found that in *P. acuminata* a large fraction of the Ni can be stored in the roots, when Ni availability is high.

Nickel transport from roots to the shoots in hyperaccumulator plants has been attributed to the xylem (Alves et al., 2011; Rascio and Navari-Izzo, 2011). Accordingly, we found that the Ni concentrations in the xylem sap of *P. acuminata* trees was particularly high (313–1,426 µg g^−1^) (**Supplementary Table 2**), implying Ni transport in the xylem. In our experiment the control treatment was devoid of Ni and Ni in seedlings, most probably derived from cotyledons, was only partially transferred to the shoot. Translocation of Ni through the phloem has been suggested to occur in hyperaccumulators (Deng et al., 2016), and was revealed by tissues-level analysis that showed high concentrations of Ni in the phloem of hyperaccumulators (Mesjasz-Przybylowicz et al., 2016; van der Ent et al., 2017). High Ni concentrations have also been found in the sap of hyperaccumulators species, but phloem sap sampling is a technically challenging task (Álvarez-Fernández et al., 2014). In plants with laticifers or other ducts, Ni could also be preferentially stored in these cells that are found in all plant tissues and sometime in the phloem itself (Evert, 2006). Whether Ni translocation, from cotyledons to leaves and from old leaves to young leaves, in *P. acuminata* occurs *via* the phloem and/or the laticifer network remains an intriguing question. Current understanding suggests that laticifers provide an effective location to store a wide array of secondary compounds, but very little is known about putative ion trafficking through laticifer networks (Hagel et al., 2008; Castelblanque et al., 2016). Translocation through laticifers has been advocated but not investigated (Kutchan, 2005).

Based on our results, we posit that at low Ni concentrations in the solution, Ni accumulated preferentially in old leaves in comparison to the apical shoot and root. At high Ni concentrations in the solution, the maximum storage capacity in roots and old leaves is reached and Ni is actively transported to the apical shoots. At the whole plant scale, this physiology would reflect a tendency for higher Ni concentrations in the shoots when bioavailable Ni is high. Interestingly, the unique whole-plant scale accumulation pattern of Ni in *P. caeruleilatex* gives insights into this physiology. *Pycnandra caeruleilatex* has latex in the main trunk and older branches that is uncolored and thus apparently Ni-poor, but the latex in the apical shoots and petioles of apical leaves is distinctly blue-green (*i.e.* Ni-rich) (**Figures 1E, G**). This [Ni] distribution pattern could reflect the dynamics of Ni transport and translocation, but more investigation in this remarkable model species is required.

### Evolution of Hyperaccumulation in *Pycnandra*

This study highlights repeated, but infrequent, evolution of hyperaccumulation within the genus *Pycnandra*, the syndrome being variously expressed in the different species. Hence, hyperaccumulation evolved at least two times within the genus, in the subgenera *Sebertia* and *Trouettia*, while it is (nearly) absent in subgenera *Achradotypus, Pycnandra*, and *Leptostylis*. The divergence between *Pycnandra* and its sister group *Niemeyera*, an Australian endemic, is estimated to 29.8 Myr (22.3–38 Myr) (Swenson et al., 2014), the genus being the oldest lineages estimated to date in the flora of New Caledonia (Pillon, 2012). In the sister genus *Niemeyera*, no species are known to occur on ultramafic soils (Atlas of Living Australia, ala.org.au), and hyperaccumulation does not occur in the genus. The genus *Pycnandra* has been estimated to have colonized the main island (Grande Terre) between 29.8–16.2 Ma (stem and crown-node age) ago (Swenson et al., 2014). Altogether, this suggests that constitutive hyperaccumulation in *Pycnandra* has evolved *in situ*, during a very long period of exposure to New Caledonian ultramafic soils. The infrequent evolution of hyperaccumulation in the genus *Pycnandra*, despite a long evolutionary time since island colonization, suggests that the syndrome might be strongly physiologically constrained and/or has a complex selective advantage. A drawback is that in *Pycnandra*, no clade is strictly associated with ultramafic soils. Instead, soil preference is labile within clades (Swenson and Munzinger, 2016). This is in accordance with the few studies, where ultramafic preference has been mapped onto phylogenies, showing its highly labile evolutionary origin (Anacker, 2011). Nickel hyperaccumulation is a syndrome of ultramafic soil that has evolved repeatedly in many clades in the flora of New Caledonia (Pillon et al., 2010; Jaffré et al., 2013; Gei et al., 2020) as elsewhere (Reeves et al., 1999; Borhidi, 2001; Krämer, 2010; Cappa and Pilon-Smits, 2014). It has, however not been shown to lead to adaptive radiation or important diversification within a clade. For example, in neotropical *Psychotria* hyperaccumulation probably occurs in only a few distantly related species (McCartha et al., 2019). Under ultramafic conditions, hyperaccumulation might maximize fitness and competitive ability but this selective advantage is subtle and depends on species physiology, environment, and probably biotic pressures. A gradient from accumulation to hyperaccumulation is found in many clades inclined to tolerate high Ni concentrations. The evolution of hyperaccumulation might thus be a gradual evolutionary process (Boyd, 2012), and this explains why it is so challenging to understand the evolution of this syndrome.

### The Evolutionary Advantage of Ni Hyperaccumulation in *P. acuminata*

It is striking that only three species with Ni-rich laticifers, and two marginal hyperaccumulators, occur in *Pycnandra*, which is the largest endemic genus of New Caledonia (Munzinger et al., 2020. [continuously updated]) and frequently occurs on ultramafic soils (Swenson and Munzinger, 2016). Several hypotheses have been put forth to explain the selective advantage of hyperaccumulation, ranging from tolerance (sequestration), disposal from the plant through leaf abscission, drought resistance, interference (allelopathy), or pathogen/herbivore defence (Martens and Boyd, 1994; Boyd and Martens, 1998; Boyd and Jaffré, 2001; Bhatia et al., 2005). The defensive enhancement hypothesis has received most research attention and is backed up with experimental evidence for some species (see Boyd, 2012 for a review). The principal roles of laticifers are in wound healing, defence against herbivory, and pathogen defence (Agrawal and Konno, 2009). The high-pressure laticifers ducts of *P. acuminata* are effectively weaponized with toxic Ni-citrate salt to which sap sucking or browsing insects are exposed, suggesting a role in elemental defence. Spectacular internal excretion (deposition) of inorganic compounds in laticifers could provide an effective way to gain defence metabolites at potentially lower cost. During field work, however, we found many fruits of *P. acuminata* infested by Apionidae larvae that reached maturity (**Figures 5C, D**), suggesting insect specialization with high levels of tolerance, and important seed predation. Several host associations between genera of Apionidae (weevil family) and Sapotaceae have already been documented, including with several species of *Pycnandra* (Wanat and Munzinger, 2012), but never with hyperaccumulator species. Herbivore interactions and Ni-tolerant insects have also been reported for *P. acuminata* fruits, whose fleshy pericarp is highly Ni-enriched (Sagner et al., 1998; Boyd et al., 2006). Although so-called "high-Ni insects" are known, community‐level studies show that bioaccumulation of Ni does not occur at the plant‐herbivore or herbivore‐predator steps. (Boyd et al., 2006; Migula et al., 2007).

A major finding of the hydroponics experiment, apart from the unreported extremely high levels of Ni tolerance, is the enhanced growth at 100 μM Ni at Ni concentrations exceeding values previously reported (Nishida et al., 2011; Iori et al., 2013; Deng et al., 2014). It is known that Ni has a stimulatory effect on some plants, but at very low prevailing concentrations (Mishra and Kar, 1974). Purely growth stimulatory effects at high Ni concentrations (100 µM) have not previously been reported, even in Ni hyperaccumulator plants. The question then arises as to why Ni has a growth promoting effect at concentrations millions of times greater than is required for urease activity (< 5 µg g^−1^ foliar Ni, Eskew et al., 1983)? Because *P. acuminata* stores a large portion of Ni in laticifer and epidermal cells (Sagner et al., 1998), only a small fraction of Ni in the plant might actually be involved in urease activity, but perhaps enough to stimulate the recycling of endogenous nitrogen and stimulate growth, at least in the first developmental stages.

The ecological significance of hyperaccumulation in *P. acuminata* could also be related to interference and allelopathy benefits. Phyto-enrichment of the litter, leading to an increase in bioavailable Ni in the soil, has been suggested to affect the germination and growth of competing plant species (Boyd and Jaffré, 2001), and may provide an ecological advantage for hyperaccumulator plants if they need more Ni than other plants. Hyperaccumulators might benefit from *in situ* (fruit and seed) high Ni concentrations at the seedlings stage and phytoenrichment from others hyperaccumulator in latter stage of growth. Chaney et al. (2009) failed to obtain proof of a functional Ni deficiency in *Alyssum* because seeds supplied enough Ni for growth, in a 6-week growth period. We believe that Ni contained in seeds plays an essential role in the seedling phase of hyperaccumulator plants, such as *P. acuminata*.

### The Incidence of Nickel-Rich Laticifers May Have Been Overlooked

One of the major physiological constrains of hyperaccumulation is the ability to store metal in high concentrations without interfering with basic plant metabolism. Such physiological constraints could limit the evolution of hyperaccumulation to few clades. The fact that *P. acuminata* (and *P. kouakouensis* and *P. caeruleilatex*) can accumulate extraordinary levels of Ni in their tissues is because of the storage capacity of laticifers (that account for a large volume in stem, leaves and roots, *unpublished data*), and this might constitute a physiological advantage for the evolution of hyperaccumulation.

Laticifers are defined as internal secretory structures for secondary metabolites (Hagel et al., 2008). Accumulation of Ni in laticifers of hyperaccumulator plants demonstrates that they might extend their physiology to extreme levels of internal excretion (deposition) of inorganic compounds for metal detoxification and possibly transport (van der Ent et al., unpublished). This “novel function” connects to the concept of exaptation *(i.e*. “function of object now being used in another way”) (Gould and Vrba, 1982).

Nickel-rich laticifers have also been described from *Euphorbia helenae* subsp. *grandifolia* (Euphorbiaceae) which has a latex with 30,900 µg g^−1^ Ni (Reeves et al., 1996) and two *Phyllanthus* species from Cuba (Berazaín et al., 2007). Another example is *Cnidoscolus* cf. *bahianus* (Euphorbiaceae) from Brazil which produces a latex that contains 13,500 µg g^−1^ Ni, whereas the leaves are much less Ni-rich with only 100 to 1,020 µg g^−1^ Ni (Reeves et al., 2007). Field qualitative testing of several other species belonging to *Euphorbia, Chamaesyce*, and *Manihot* also revealed latex containing >1,000 µg g^−1^ Ni (Reeves et al., 2007). Observation of Ni-rich latex have also been made in *Planchonella roxburghiana* (Sapotaceae) and *Ficus trachypison* (Moraceae) from Halmahera (Indonesia) (Lopez et al., 2019). Hyperaccumulator plants with Ni-rich laticifers appear to be rare in the plant kingdom, but the phenomenon could have been largely overlooked. Many Ni hyperaccumulator plant species are in families known to produce laticifer cells (*e.g.* Asteraceae, Phyllanthaceae, Euphorbiaceae, Metcalfe, 1967) suggesting that more investigations into this phenomenon may be warranted.

## Conclusion

Our study reveals outstanding Ni tolerance in a woody hyperaccumulator that is related to the plant scale distribution and large internal volume of laticifer cells. We also showed that growth is stimulated by Ni concentrations in the solution as high 100 μM Ni and Ni in the seed store of the cotyledons is translocated to the young shoots. These results strongly suggest a higher Ni requirement of this species, although the underlying function(s) remain elusive. Our study relied on a rare species, but the observation of Ni-rich latex in other hyperaccumulators species, suggest that this mechanism of Ni storage might be more common than previously thought. Future research is needed to elucidate how laticifer networks are involved in Ni trafficking throughout the plant.

## Data Availability Statement

The raw data supporting the conclusions of this article will be made available by the authors, without undue reservation.

## Author Contributions

AE and SI conceived the research. SI, VG, PE, and AE collected the plant samples in New Caledonia. LL’H performed the hydroponic experiments. VG conducted the XRF screening. AP and AE conducted the chemical analysis of plant tissue samples; JM made the matrix for the phylogenetic tree. SI, LL’H, and AP analyzed the data. SI and AE wrote the article with contributions from all authors. SI is the corresponding author for this work.

## Funding

AE was the recipient of a Discovery Early Career Researcher Award (DE160100429) from the Australian Research Council. VG was the recipient of an Australia Awards PhD Scholarship from the Australian Federal Government. This work was supported by UMR AMAP.

## Conflict of Interest

The authors declare that the research was conducted in the absence of any commercial or financial relationships that could be construed as a potential conflict of interest.
