# Supplementary material for: Novel Insights Into the Hyperaccumulation Syndrome in Pycnandra (Sapotaceae)
Source: Front Plant Sci. 2020 Sep 9;11:559059. doi: 10.3389/fpls.2020.559059 (PMC7509057; doi:10.3389/fpls.2020.559059)
Supplement: Supplementary file 1 [file Table_1.docx]

Supplementary Material

**1. Herbarium X-ray Fluorescence Spectroscopy: Method and calibration**

A Thermo Fisher Scientific Niton XL3t 950 GOLDD+ instrument was used to measure the foliar elemental concentrations of the herbarium specimens (Gei et al. The instrument contains a miniature X-ray tube (Ag anode (6–50 kV, 0–200 µA max)), with a geometrically optimized large area silicon drift detector (SDD) {van der Ent, 2019 #2602}. It can detect elements from Mg to U within 15–60 seconds with detection limits ~50–100 µg g^-1^, and for most elements the detection limit is 50 µg g^-1^. Dried herbarium leaf specimens were subjected to an incident beam of X-rays for 30 seconds in the ‘Soils Mode’ (which uses Compton Normalization). This new calibration was obtained from 221 specimens from the Herbarium of New Caledonia (NOU) that were intentionally chosen to cover a very wide concentration range (low range to hyperaccumulation range for Mn, Co, Ni, Zn) on the basis of the earlier study. From each specimen a 1 cm^2^ area was destructively excised from each specimen, analysed by XRF and after digestion by ICP-AES. The resulting regression equation used for ‘calibration’ were substantially improved (for instance R^2^ for improved from 0.87 to 0.98), and the newer calibration was used in this study. In total, 2131 specimens of the Sapotaceae, including 847 specimens of *Pycnandra*, were measured.

The apparent limits of detection (LOD) for Co, Ni, Mn and Zn were estimated by visual inspection of the log-transformed regression models of the XRF data against corresponding ICP-AES measurements and set at XRF values: 420 μg g^-1^ for Co, 455 μg g^-1^ for Mn, 110 μg g^-1^ for Ni and 30 μg g^-1^ for Zn (range 27–1238, n = 117). The regression models (y = calculated ICP-AES; x = measured XRF) are: Co: y = 0.429x^0.9809^ (R^2^ 0.92; n=50), Mn y = 0.7869x^0.9165^ (R^2^ 0.98; n=159), Ni: y = 0.2351x^1.0969^ (R^2^ 0.98; n=149) and Zn: y = 0.3766x^1.1259^ (R^2^ 0.88; n=117).

**Supplementary Figure 1** Calibration plots for calculation of foliar concentrations for each trace element (Ni, Mn, Co, Zn).

| A | 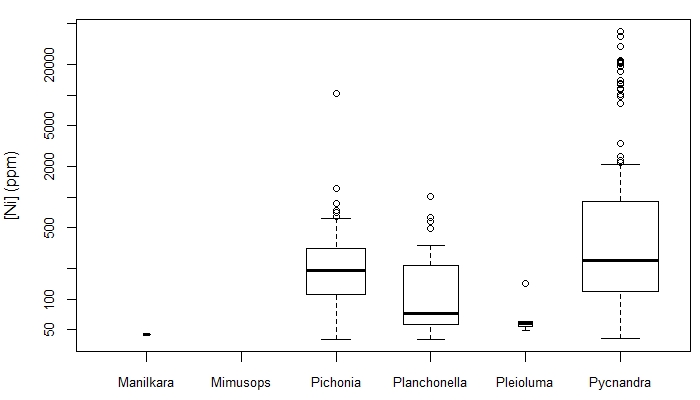 | | |
| --- | --- | --- | --- |
| B | | 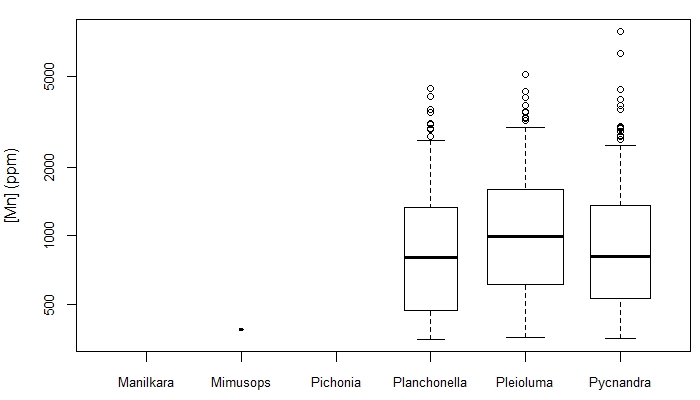 |  |
| C | 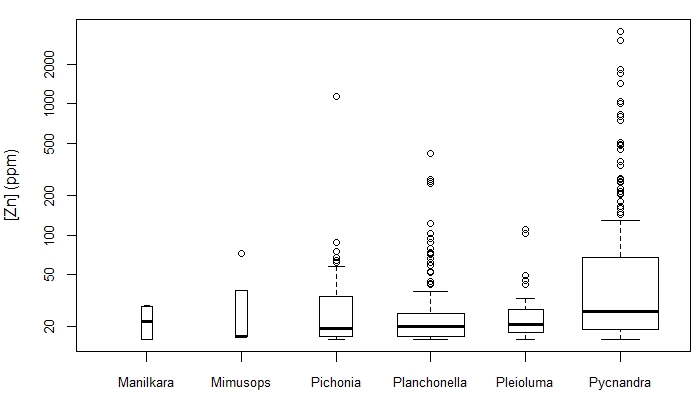 | | |

**Supplementary Figure 2** Boxplots of Ni (A), Mn (B) and Zn (C) foliar concentrations (ppm = µg g^-1^) measured on the six genera of Sapotaceae occurring in New Caledonia. Data were obtained from Herbarium X-Ray fluorescence (XRF) scanning undertaken on all specimens (n=2131) from the Herbarium of New Caledonia (NOU, IRD/Nouméa). The dashed red line indicates the hyperaccumulation threshold for each element. None of the measured specimens had Co concentration values above the limit of detection.

**Supplementary Table 1.** Bulk elemental concentrations (ICP-AES) in *Pycnandra caeruleilatex* and *Pycnandra kouakouensis* (values as ranges and means in µg g^-1^ dry weight).

| **Elemental concentrations** | | | | | | | | | | | |
| --- | --- | --- | --- | --- | --- | --- | --- | --- | --- | --- | --- |
| **Plant species** | **Plant tissue** | **n** | **Mg** | **P** | **K** | **Ca** | **Mn** | **Fe** | **Co** | **Ni** | **Zn** |
| ***Pycnandra kouakensis*** |  |  |  |  |  |  |  |  |  |  |  |
|  | Latex | 1 | 2320 | 52.8 | 750 | 21 100 | 1270 | 76.1 | 21.2 | 124 000 | 695 |
|  |  |  |  |  |  |  |  |  |  |  |  |
|  | Leaves | 2 | 990  (870–1110) | 257  (193–321) | 4440  (2570–6300) | 2340  (1920–2760) | 42.3  (39.6–44.9) | 19.2  (2.09–36.3) | 5.43  (0.76–10.1) | 3610  (1150–6080) | 39.7  (10.1–69.3) |
| ***Pycnandra caeruleilatex*** |  |  |  |  |  |  |  |  |  |  |  |
|  | Apical tip | 1 | 2000 | 551 | 10 800 | 13 300 | 90.2 | 31.3 | 0.82 | 2470 | 34.0 |
|  |  |  |  |  |  |  |  |  |  |  |  |
|  | Phloem | 1 | 680 | 140 | 2540 | 68 600 | 51.3 | 88.0 | 1.17 | 770 | 40.0 |
|  | Wood | 3 | 820  (670–1060) | 139  (132–147) | 2730  (1940–3270) | 21000  (17000–27700) | 49.0  (41.1–64.0) | 60.5  (26.4–84.2) | 0.64  (0.51–0.81) | 1270  (1090–1370) | 46.8  (32.1–55.5) |

**Supplementary Table 2.** Literature survey of Ni concentration in xylem sap of hyperaccumulators species, and Ni concentration in xylem sap of *P. acuminata* measured in this study.

| **Species** | **Distribution** | **Fluid** | **Ni concentration** | **Reference** |
| --- | --- | --- | --- | --- |
| *Alyssum corsicum* | SE Europe | Xylem sap | 3250 μM | Centofanti et al. 2013 |
| *Alyssum serpyllifolium* | Iberian Peninsula | Xylem sap | 950 μM | Alves et al. 2011 |
| *Cistus ladanifer* | Iberian Peninsula | Xylem sap | 170 μM | Lázaro et al. 2006 |
| *Flacourtia kinabaluensis* | Borneo, SE Asia | Xylem sap | 51 μM | van der Ent and Mulligan 2015 |
| *Phyllanthus balgooyi* | Borneo and Palawan Islands, SE Asia | Xylem sap | 1290 μM | van der Ent and Mulligan 2015 |
| *Phyllanthus rufuschaneyi* | Borneo and Mindanao, SE Asia | Xylem sap | 221 μM | van der Ent and Mulligan 2015 |
| *Psychotria* cf*. gracilis* | Borneo, SE Asia | Xylem sap | 834 μM | van der Ent and Mulligan 2015 |
| ***Pycnandra acuminata*** | **New Caledonia** | **Xylem sap** | **13 900 μM** | **This study** |
| *Rinorea* cf*. bengalensis* | SE Asia | Xylem sap | 324 μM | van der Ent and Mulligan 2015 |
|  |  |  |  |  |

**References**

Alves S, Nabais C, Simoes Goncalves Mde L, Correia Dos Santos MM. 2011. Nickel speciation in the xylem sap of the hyperaccumulator *Alyssum serpyllifolium* ssp. *lusitanicum* growing on serpentine soils of northeast Portugal. *Journal of Plant Physiology* 168(15): 1715-1722.

Centofanti T, Siebecker MG, Chaney RL, Davis AP, Sparks DL. 2012. Hyperaccumulation of nickel by *Alyssum corsicum* is related to solubility of Ni mineral species. *Plant and Soil* 359(1-2): 71-83.

Deng T-H-B, Tang Y-T, van der Ent A, Sterckeman T, Echevarria G, Morel J-L, Qiu R-L. 2016. Nickel translocation via the phloem in the hyperaccumulator Noccaea caerulescens (Brassicaceae). *Plant and Soil* 404: 35-45.

Díez Lázaro J, Kidd PS, Monterroso Martínez C. 2006. A phytogeochemical study of the Trás-os-Montes region (NE Portugal): Possible species for plant-based soil remediation technologies. *Science of the Total Environment* 354(2): 265-277.

van der Ent A, Mulligan D. 2015. Multi-element Concentrations in Plant Parts and Fluids of Malaysian Nickel Hyperaccumulator Plants and some Economic and Ecological Considerations. *Journal of Chemical Ecology* 41(4): 396-408.
